# Relaxin inhibits renal fibrosis and the epithelial-to-mesenchymal transition via the Wnt/β-catenin signaling pathway

**DOI:** 10.1080/0886022X.2022.2044351

**Published:** 2022-03-21

**Authors:** Chen Feiteng, Chen Lei, Li Deng, Xu Chaoliang, Xu Zijie, Shao Yi, Sha Minglei

**Affiliations:** a Department of Urology, Shanghai General Hospital, Shanghai Jiao Tong University School of Medicine, Shanghai, China; b Department of Geriatric, Shanghai General Hospital, Shanghai Jiao Tong University School of Medicine, Shanghai, China

**Keywords:** Renal fibrosis, chronic kidney disease, epithelial-to-mesenchymal, unilateral ureteral obstruction

## Abstract

Renal fibrosis is a common characteristic and the final pathological mechanism of chronic kidney disease (CKD). Although CKD remains incurable, inhibition of renal fibrosis is beneficial to inhibit the CKD process. Relaxin alleviates renal fibrosis in some experimental models, but its mechanism remains unclear. In the following, we studied the regulatory effect of relaxin on epithelial-mesenchymal transition (EMT) after unilateral ureteral obstruction (UUO). Our results demonstrate that relaxin could downregulate Wnt/β-catenin signaling and decrease EMT, thus protecting against loss of transporters in tubular epithelial cells (TECs) and abrogate renal interstitial fibrosis following UUO. We confirmed that relaxin can downregulate Wnt/β-catenin signaling and decrease EMT in NRK52E, thus abrogating G2 cell cycle arrest *in vitro* experiments. Therefore, a novel mechanism by which relaxin is antifibrotic is that relaxin regulates the EMT program of TECs *via* Wnt/β-catenin signaling pathway. The inhibition of EMT contributes to protecting the functional capabilities of TECs and promoting the regeneration of TECs.

## Introduction

CKD is one of the leading causes of death worldwide and is prevalent in many countries, and the incidence of CKD is on the rise for which there is currently no effective treatment [1,2]. Renal fibrosis, characterized by a progressive decline of renal function and loss of renal parenchyma, is the common final pathological pathway of almost all CKDs, which eventually leads to impaired renal function and final end-stage renal failure, requiring patients to rely on lifelong dialysis or renal transplantation [3,4]. It is, therefore, necessary to explore the complex molecular and cellular mechanisms of renal fibrosis, which may provide new therapeutic strategies.

The transition of TECs into cells with mesenchymal features, a so-called EMT feature, is an important feature of renal fibrosis [5–8]. EMT involves dramatic changes in epithelial cell morphology and behavior, such as loss of epithelial adhesion, cytoskeletal reorganization and *de novo* synthesis of a-SMA, disruption of the tubular basement membrane, and ultimately enhanced cell migration and mesenchymal invasion [9]. Recently, it was reported that the process of EMT triggers G2 cell cycle arrest [10]. G2 cell cycle arrested TECs increase collagen expression and acquire a profibrotic phenotype by increasing the expression of cytokines responsible for enhancing proliferation and collagen production of fibroblasts (TGF-β1 and connective tissue growth factor, CTGF) [11]. Based on these findings, we speculated that inhibition of EMT may be a novel target for anti-fibrosis.

Relaxin, a peptide hormone, has emerged as a fast-acting but safe anti-fibrotic drug that effectively attenuates the development of renal fibrosis in multiple experimental models, such as renal ischemia/regeneration perfusion injury model and unilateral ureteral obstruction (UUO) [12–15]. In clinical evaluation, the vasodilator function of relaxin in acute heart failure has been well studied, but its anti-fibrotic functional mechanism has not been perfected [16]. Recent studies show that relaxin inhibits TGF-β activity in human and rat kidney myofibroblasts through its cognate G-protein-coupled receptor relaxin family peptide receptor 1 (RXFP1), thereby activating neuronal nitric oxide (NO) synthase (nNOS)-NO-cyclic guanosine monophosphate (cGMP)–a dependent pathway and extracellular signal-regulated kinase phosphorylation (pERK)1/2 [17,18]. Therefore, this inhibited TGF-β-induced myofibroblast-derived abnormal matrix/collagen production and myofibroblast differentiation, while increasing matrix metalloproteinases, which can break down collagen. While considering that TGF-β is the inducer of EMT, we hypothesized that relaxin could decrease EMT and cell cycle arrest in TECs, thereby reducing the incidence of renal fibrosis after UUO. Our results suggest that relaxin downregulates Wnt/β-catenin signaling and decreases EMT, thus protecting TEC transporters and abrogating renal interstitial fibrosis following UUO. *In vitro* studies proved that relaxin can downgrade β-catenin signaling and decrease EMT in NRK52E, thus abrogating G2 cell cycle arrest.

## Materials and methods

### Animal model

C57BL/6 male mice were provided by our institute’s laboratory animal center. Ethical approval for the animal study was granted by the Institutional Animal Care and Use Committee at Shanghai First People's Hospital of Shanghai Jiao Tong University. The Ethics Committee of Shanghai General Hospital approved all animal experiments (Permit Number: 2019-A104-01).

From 2 days before UUO to 5 days after injury, mice were prophylactically injected with recombinant H2 relaxin (PeproTech, Rocky Hill, NJ, USA) (0.5 mg/kg per day) *via* subcutaneously implanted osmotic minipumps (model 1007 D; Alzet, Cupertino, CA, USA). Regardless of etiology, relaxin at this dose has been previously used to successfully prevent or reverse fibrosis progression in various models of kidney disease [13].

We adapted a well-established UUO model of renal fibrosis to study interstitial fibrosis. The animals were intraperitoneally anesthetized with Zoletil50. After that, the right ureter of mice was ligated, and the left kidney remained intact. Exposed right ureter after a midline abdominal incision. The mid-ureter was blocked by a two-point ligation with silk sutures. Sham-operated mice underwent the same procedure except for ureteral obstruction. Close the abdominal incision and allow the mouse to recover. The kidneys were harvested 5 days after surgery.

### Cell culture and differentiation

NRK52E (ATCC, Manassas, VA, USA) cell line was cultured in a DMEM medium (Hyclone, Logan, UT). The cells were maintained at 37 °C under 5% CO2 in a humidified chamber. After incubation in the medium containing 1% FBS for 24 h, cells were treated with 10 ng/ml Recombinant Human TGF-β1 (Peprotech, NJ, USA) alone, 100 ng/ml relaxin alone or co-treated with TGF-β1 and relaxin for 48 h. To induce the Wnt/β-catenin pathway, NRK52E cells were treated with 40 μM SKL2001 (MedChemExpress LLC, Monmouth Junction, NJ, USA).

### Histological analysis

We performed Sirius red staining using formalin-fixed, paraffin-embedded kidney sections to assess the extent of collagen deposition. Sirius Red staining was performed using the Picro-Sirius Red Stain Kit(Abcam). Quantification was performed with ImageJ (Nation Institutes of Health).

### Immunohistochemistry

Kidneys 5 days after UUO were retrieved, fixed in 10% formalin, embedded in paraffin, and cut into 8-μm sections. Immunohistochemical staining was performed on paraffin-embedded sections [19]. Primary antibodies used were vimentin (5741 P, CST), E-cadherin (20874-1-AP, ProteinTech), AQP1 (sc-25287, Santa Cruz), Na^+^/K^+^-ATPase α (H-3) (sc-48345, Santa Cruz), SLC22A6 (ab131087, Abcam), and secondary antibody (D-3004, Long Island Biotech, Shanghai, China). For the above immunohistochemical staining, random 5 high-power fields were acquired using a Leica DM5500 B microscope (Leica Microsystems CMS GmbH, Wetzlar, Germany) with an original magnification of 40x and processed by Adobe Photoshop CS5 software (Adobe Systems Incorporated., San Jose, California, USA) software, quantification by two blinded experiments staff alone.

### Immunofluorescence staining

Protein expression and localization were examined by *in situ* immunofluorescence staining. Kidney sections were deparaffinized and hydrated overnight at 4 °C with primary antibodies (anti-p-H3 (Ser10) Abcam, Cambridge, UK; anti-Ki67 Abcam, Cambridge, UK). After 3 washes with PBS, sections were incubated with Alexa Fluor 488 or 588 (Molecular Probes, Inc., Eugene, OR, USA)-conjugated secondary antibodies for 2 h at room temperature. Then sections were counterstained with DAPI to visualize the nuclei. Taken images with the confocal microscope (Olympus, Tokyo, Japan).

### Quantitative RT-PCR (qRT-PCR)

RNA was isolated from harvested cells and kidneys using the Trizol total RNA extraction kit (Invitrogen, Carlsbad, CA, USA). 1 µg RNA was reverse transcribed using PrimeScript^TM^ RT reagent kit (Takara, Otsu, Japan). QRT-PCR was run using the SYBR Premix Ex Taq™ II kit (Takara, Otsu, Japan) and gene-specific primers. The expression levels were calculated using the 2^-ΔΔCT^ method and were normalized and analyzed to GAPDH levels. Real-time primer sequences used in the present study were shown in Table 1.

**Table 1.** Primers for real-time PCR analysis.

Gene	Primer sequence (5′- 3′) (mice)	Primer sequence (5′- 3′) (NRK52E)
GAPDH Fwd	ATCATCCCTGCATCCACT	GGTGGACCTCATGGCCTACA
GAPDH Rew	ATCCACGACGGACACATT	CTCTCTTGCTCTCAGTATCCTTGCT
Vimentin Fwd	CTTGAACGGAAAGTGGAATCCT	TCGCCAACTACATCGACAAG
Vimentin Rew	GTCAGGCTTGGAAACGTCC	TCCCTCATCTCCTCCTCGTA
E-cadherin Fwd	GCA GTT CTG CCA GAG AAA CC	GGTCTCTTGTCCCTTCCACA
E-cadherin Rew	TGG ATC CAA GAT GGT GAT GA	CTCCAGACCCACACCAAAGT
β-catenin Fwd	TAAACTCCTGCACCCACCAT	CTTACGGCAATCAGGAAAGC
β-catenin Rew	GTCGTGGAATAGCACCCTGT	TCAGCACTCTGCTTGTGGTC
Twist Fwd	CTGCCCTCGGACAAGCTGAG	GTCGCTGAACGAGGCATT
Twist Rew	CTAGTGGGACGCGGACATGG	GGACCTGGTACAGGAAGTCG
Snail Fwd	CACACGCTGCCTTGTGTCT	CGTGTGTGGAGTTCACCTTC
Snail Rew	GGTCAGCAAAAGCACGGTT	CCAGGAGAGAGTCCCAGATG
AQP1 Fwd	AGGCTTCAATTACCCACTGGA	
AQP1 Rew	GTGAGCACCGCTGATGTGA	
ATP1B1 Fwd	TCGGGACCATCCAAGTAA	
ATP1B1 Rew	TCGGGACCATCCAAGTAA	
SLC22A6 Fwd	CTGATGGCTTCCCACAACAC	
SLC22A6 Rew	GTCCTTGCTTGTCCAGGGG	

### Western blotting analysis

Total protein was extracted in cells or kidney tissue using RIPA (Beyotime Biotechnology, Shanghai, China) and quantified using the BCA (Thermo Scientific, Waltham, MA, USA) assay kit. Equal amounts of protein from each sample were electrophoresed on 10% Tris-glycine sodium dodecyl sulfate-polyacrylamide gels, and western blots were transferred to 0.45-mm polyvinylidene fluoride membranes (Millipore, Darmstadt, Germany). Membranes were blocked with TBST containing 5% nonfat dry milk for one hour at room temperature and then incubated with primary antibodies against mouse fibronectin (ab45688, Abcam), vimentin (5741 P, CST), N-cadherin (22018-1-AP, ProteinTech), β-catenin (ab32572, Abcam), snail (3879 P, CST), twist (sc-81417, Santa Cruz) or GAPDH (#5174, CST) overnight at 4 °C, followed by incubating with a horseradish peroxidase-conjugated secondary antibody. Densitometric analysis results were standardized as GAPDH expression and expressed as fold change relative to those of negative control.

### Data analysis

All statistical analyses were run using SPSS (version 19.0.0, SPSS Inc., Chicago, IL, USA). The results are shown as the means ± SD of at least three independent experiments. Two-sided Student’s test-test or one-way ANOVA were used to analyze the differences between groups. *p* < 0.05 was set as statistically significant.

## Result

### Relaxin attenuated fibrosis following UUO

**Figure 1.**
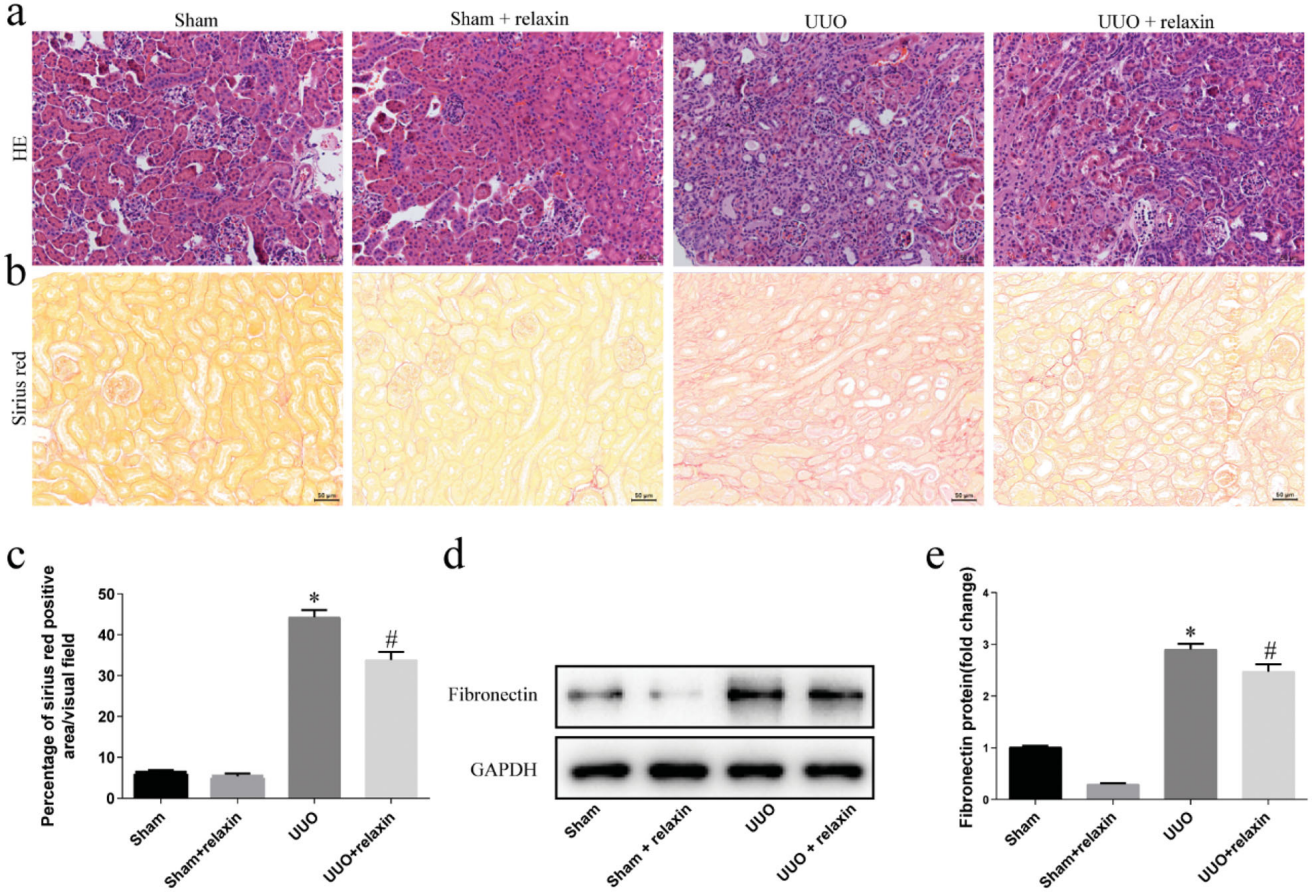
Relaxin alleviated fibrosis following UUO. (a,b) Representative images of H&E (a) and Sirius red (b) staining in the kidneys from the indicated experimental groups. Scale bars, 50 µm. (c) Interstitial fibrosis based on Sirius red staining. (d–e) Whole kidney lysates were prepared from the kidneys of the sham, sham + relaxin, UUO and UUO + relaxin mice and analyzed for changes in fibrosis (fibronectin) by western blotting. The expression of the indicated proteins in the kidneys was analyzed by densitometry, normalized to the GAPDH levels and presented as means ± standard errors of the means (s.e.m.) *p < 0.05 vs sham, #*p* < 0.05 vs UUO.

To determine the function of relaxin in renal fibrosis, we pretreated mice with relaxin (0.5 mg/kg per day) or vehicle and analyzed them on day 5 after UUO using H&E, Sirius Red staining, and expression of fibronectin to evaluate the degree of renal fibrosis. 5 days after UUO in the early stage, when renal fibrosis has already occurred, so we evaluated their kidneys at this time. Obvious interstitial inflammation and fibrosis were observed 5 days after UUO operation and relaxin could reduce interstitial fibrosis and cell infiltration according to the result of H&E and Sirius red staining (Figure 1(a,b)). Kidneys of vehicle-treated mice had higher fibronectin levels compared to the mice pretreated with relaxin (Figure 1(d)). The result showed that relaxin can attenuate UUO-induced renal fibrosis.

### Relaxin attenuated EMT following UUO

**Figure 2.**
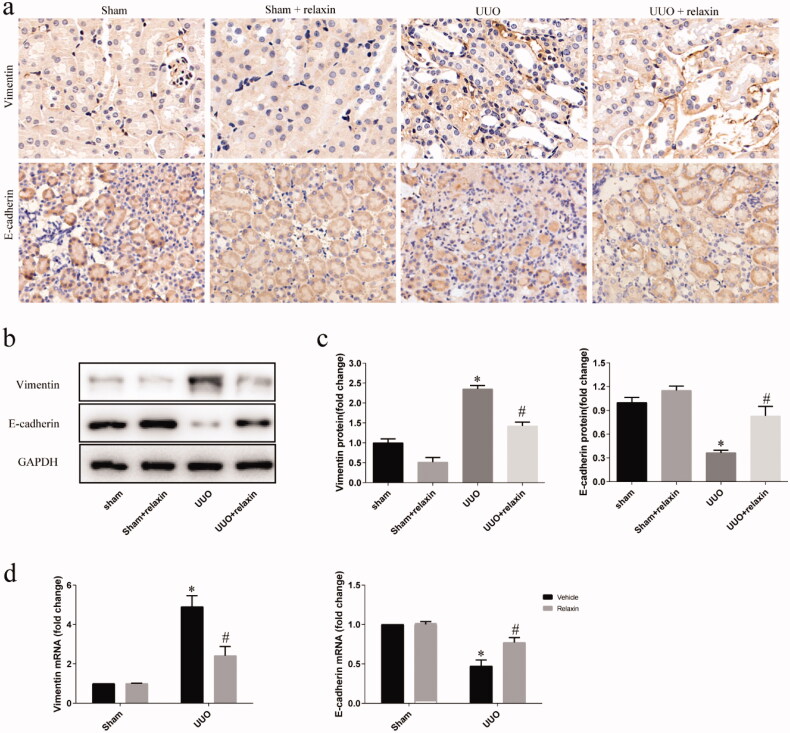
Relaxin attenuated the UUO-induced EMT. (a) Representative images (eight visual fields for each tissue sample analyzed) of vimentin and E-cadherin immunolabeling in the indicated experimental groups. The original magnification was 40×. (c–e) Whole kidney lysates were prepared from the kidneys of the sham, sham + relaxin, UUO and UUO + relaxin mice and analyzed for changes in the levels of the vimentin and E-cadherin proteins and mRNAs by western blotting (c, d) and qRT-PCR (e), respectively. The expression of the indicated proteins in the kidneys was analyzed by densitometry, normalized to GAPDH and expressed as means ± s.e.m. *p < 0.05 vs sham, #*p* < 0.05 vs UUO. The expression of the vimentin and E-cadherin mRNAs in the kidneys was normalized to GAPDH expression and presented as means ± s.e.m. *p < 0.05 vs sham, #*p* < 0.05 vs UUO.

In order to determine whether relaxin can affect EMT after UUO, we examined the variation in the expressions of vimentin and E-cadherin. Immunohistochemistry revealed increased labeling intensity of vimentin in the UUO compared to that in the sham-operated control kidneys and relaxin treated sham-operated kidneys. On the contrary, the intensity of E-cadherin in the UUO kidney was lower than that in the control kidney and sham-operated kidney treated with relaxin. Relaxin treatment reversed the changes in vimentin and E-cadherin (Figure 2(a)).

The protein expression of vimentin was upregulated in the UUO kidneys. Relaxin treatment significantly decreased the change in the expression of renal vimentin in UUO. On the contrary, in UUO, the protein level of E-cadherin decreased and increased significantly by relaxin treatment (Figure 2(b,c)). Similarly, real-time PCR revealed increased expressions of vimentin mRNA and decreased expression of E-cadherin mRNA in the UUO kidneys compared to that of the control kidneys. The treatment of relaxin significantly inhibited the downregulation of E-cadherin and upregulation of vimentin mRNA expression following UUO (Figure 2(d)).

### Relaxin attenuated TGF-β1-induced EMT *in vitro*

**Figure 3.**
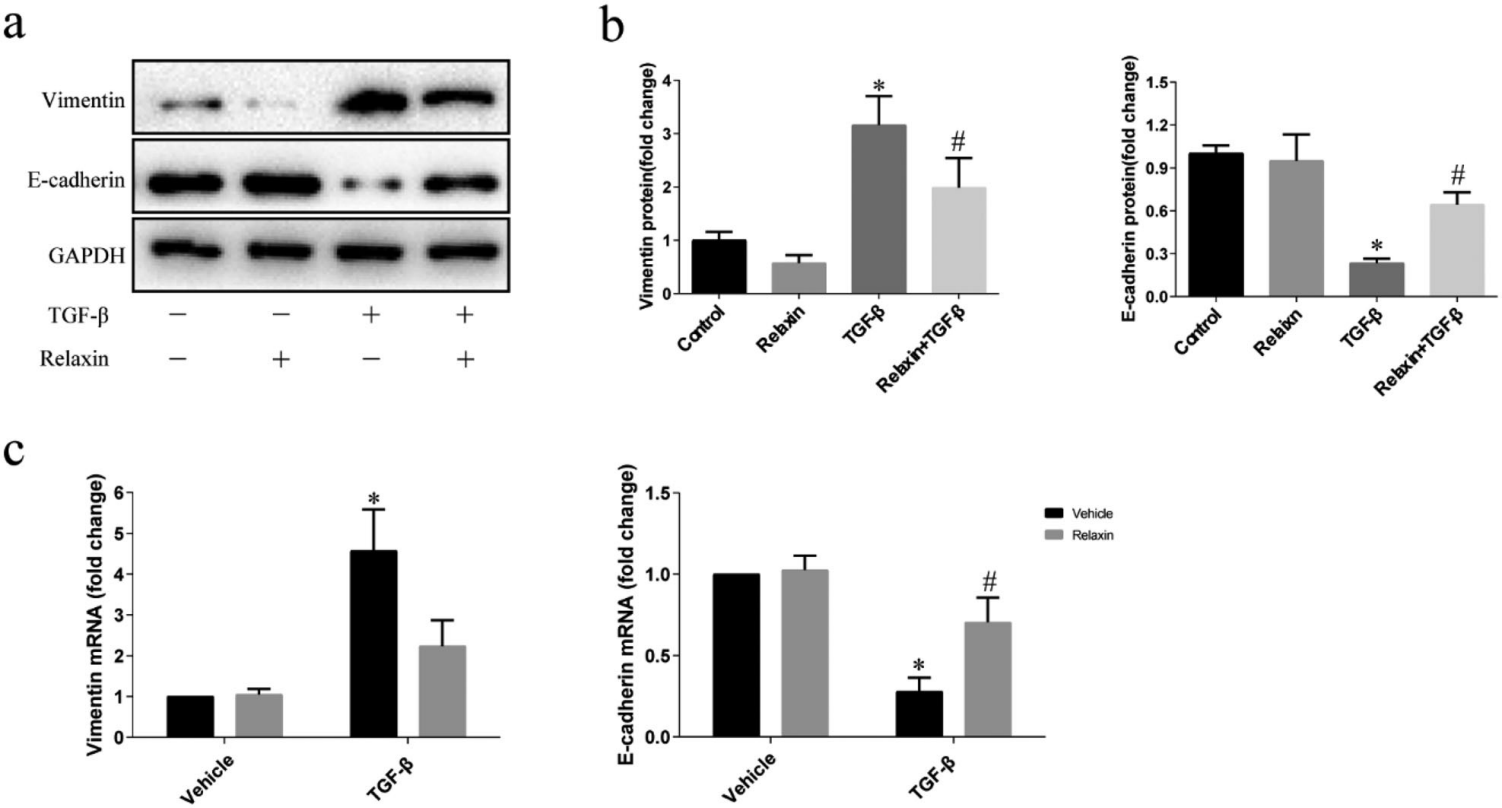
Relaxin attenuated the TGF-β1-induced EMT *in vitro*. After a 24-h incubation in a medium containing 1% FBS, NRK52E cells were treated with blank vehicle, 10 ng/ml TGF-β1 alone, 100 ng/ml relaxin alone or both TGF-β1 and relaxin for 48 h (a–c) and then the expression of the vimentin and E-cadherin proteins and mRNAs was analyzed by western blotting (a,b) and qRT-PCR (c), respectively. The gene and protein expression levels were normalized to the GAPDH levels, analyzed, and presented as means ± s.e.m; *p < 0.05 vs control, #*p* < 0.05 vs TGF-β1.

To evaluate whether relaxin could protect against TGF-β1 mediated EMT, we examined the effect of relaxin on the regulation of EMT markers in normal rat kidney proximal tubular epithelial cells (NRK52E) cells after inducing EMT by TGF-β1. After incubation in a medium containing 1% FBS for 24 h, NRK52E cells incubated with blank vehicle, 10 ng/ml TGF-β1 alone, 100 ng/ml relaxin alone, or co-treated with TGF-β1 and relaxin for 48 h. TGF-β1 treatment for 48 h upregulated the mesenchymal marker vimentin expression and downregulated the epithelial marker E-cadherin expression both in protein and mRNA level. Relaxin treatment significantly ameliorated the TGF-β1 induced downregulation of E-cadherin and upregulation of vimentin (Figure 3).

### Relaxin downregulated Wnt/β-catenin signaling pathway *in vivo* following UUO

**Figure 4.**
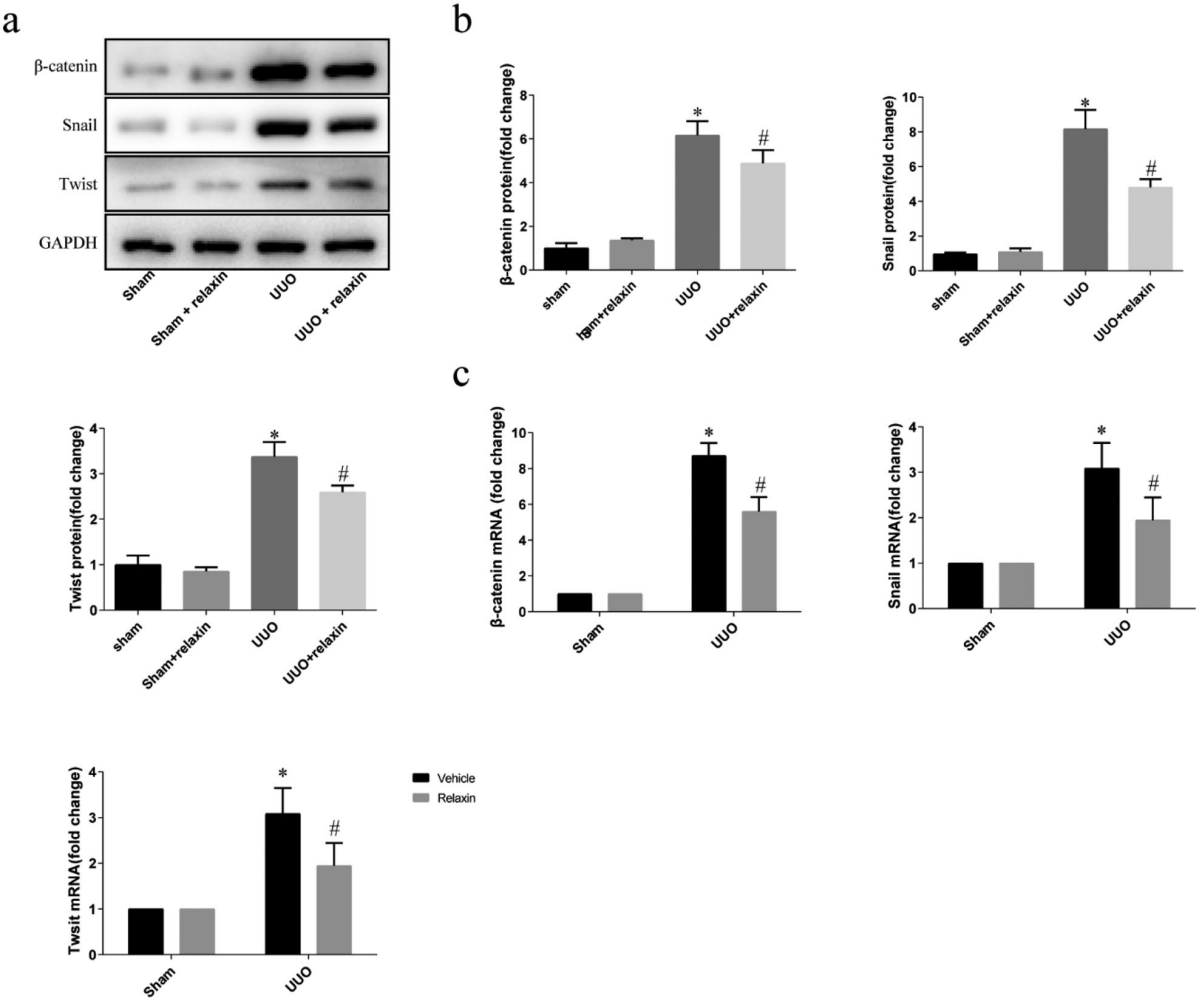
Relaxin downregulated the Wnt/β-catenin signaling pathway *in vivo* following UUO. (a,b) Whole kidney lysates were prepared from the kidneys of the sham, UUO, sham + relaxin and UUO + relaxin mice and analyzed for changes in the expression of the β-catenin, snail and twist proteins and mRNAs by western blotting (a, b) and qRT-PCR (c), respectively. The gene and protein expression levels were normalized to the GAPDH levels, analyzed, and presented as means ± s.e.m; *p < 0.05 vs sham, #*p* < 0.05 vs UUO.

Given the significance of Wnt/β-catenin signaling pathway for the EMT, and β-Catenin plays a key role in the canonical Wnt pathway, we investigated the effect of relaxin on the regulation of Wnt/β-catenin signaling pathway *in vivo* following UUO. The levels of β-catenin in kidneys were analyzed by western blot and real-time PCR. UUO kidneys showed a significant increase both in protein and mRNA level of β-catenin. Relaxin treatment significantly attenuated the upregulation of β-catenin (Figure 4). Considering that zinc-finger transcription factors play a pivotal role in EMT regulation, and there are complex crosstalks between the Wnt pathway and zinc-finger transcription factors including snail and twist [20,21], we examined the expression of snail and twist in mRNA and protein levels *in vivo* following UUO. The protein and mRNA level of snail increased in UUO. Although there was no difference in the expression of snail protein, the expression of snail mRNA was decreased significantly by relaxin treatment following UUO. The protein and mRNA level of twist increased in UUO and decreased significantly by relaxin treatment (Figure 4).

### Relaxin attenuated TGF-β1-induced EMT by modulating Wnt/β-catenin signaling pathway *in vitro*

**Figure 5.**
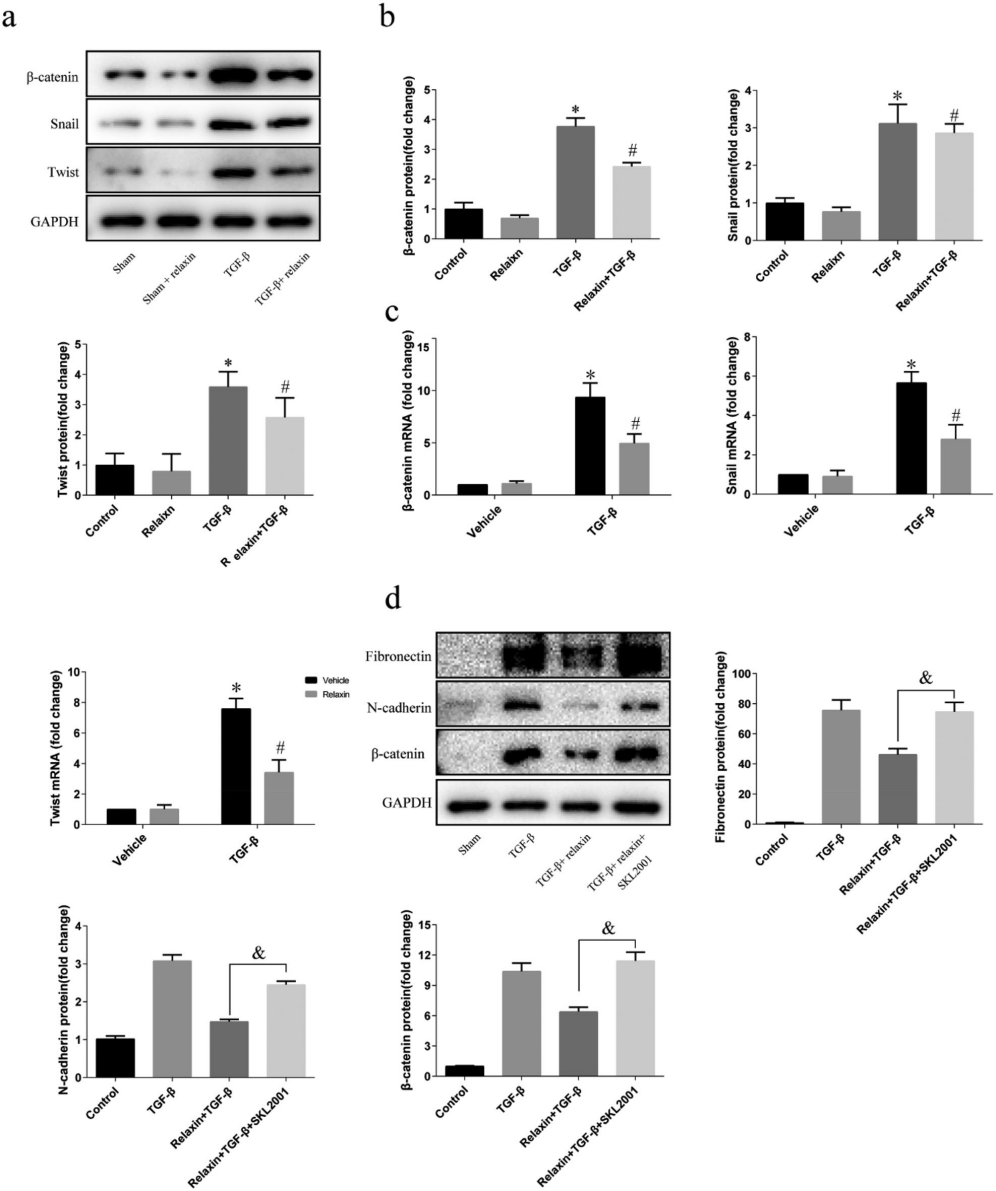
Relaxin attenuated TGF-β1-induced EMT by modulating the Wnt/β-catenin signaling pathway *in vitro*. After a 24-h incubation in a medium containing 1% FBS, the NRK52E cells were treated with blank vehicle, 10 ng/ml TGF-β1 alone, 100 ng/ml relaxin alone or both TGF-β1 and relaxin for 48 h (a–c), and the expression of the β-catenin, snail and twist proteins and mRNAs was analyzed by western blotting (a,b) and qRT-PCR (c), respectively. (d) the NRK52E cells were treated with blank vehicle, 10 ng/ml TGF-β1 alone, both TGF-β1 and 100 ng/ml relaxin or TGF-β1 + relaxin + 40 μM SKL2001 for 48 h, the expression of fibronectin, N-cadherin and β-catenin was analyzed by western blotting. The gene and protein expression levels were normalized to the GAPDH levels, analyzed, and presented as means ± s.e.m; *p < 0.05 vs control, #*p* < 0.05 vs TGF-β1, &*p* < 0.05 vs UUO + relaxin.

Considering that relaxin downregulates Wnt/β-catenin signaling pathway *in vivo* following UUO, as we described previously in this study, we then verify that relaxin can regulate the β-catenin signaling pathway *in vitro*. NRK52E cells incubated with blank vehicle, 10 ng/ml TGF-β1 alone, 100 ng/ml relaxin alone or co-treated with TGF-β1 and relaxin for 48 h after incubation in medium containing 1% FBS for 24 h. TGF-β1 treatment for 48 h upregulated β-catenin, snail, and twist expression both in protein and mRNA level. Relaxin treatment significantly attenuated the TGF-β1 induced upregulation of β-catenin, snail, and twist (Figure 5(a–c)). To further confirm that Relaxin regulates EMT through the Wnt/β-catenin pathway, we performed rescue experiments in the NRK52E cell line by using SKL2001, an activator specific for the Wnt/β-catenin pathway. As shown in Figure 5(d), SKL2001 rescued the suppression of genes caused by relaxin. Together, these results suggest that relaxin regulates EMT by targeting the Wnt/β-catenin pathway.

### Relaxin prevented loss of TEC transporters following UUO

**Figure 6.**
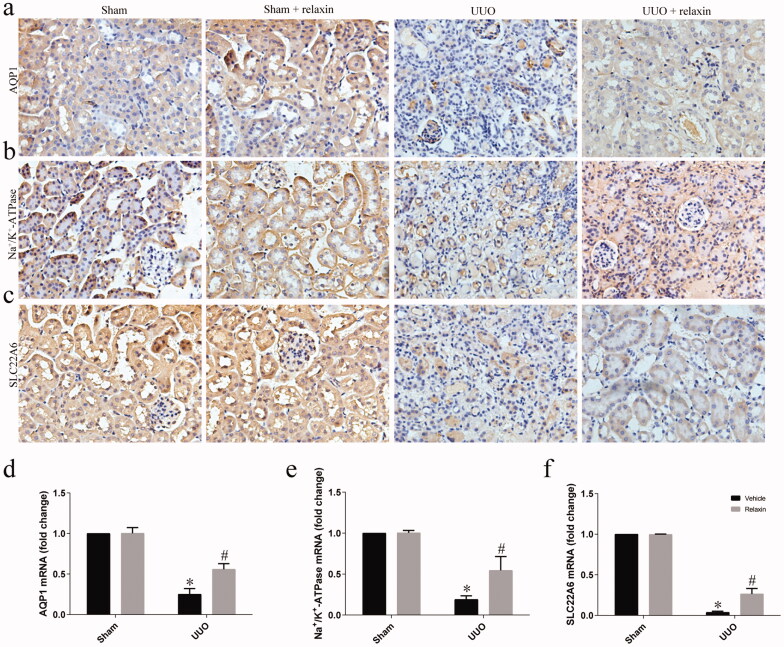
Relaxin prevented the loss of TEC transporters following UUO. (a–c) Representative images (eight visual fields for each tissue sample analyzed) of Na^+^/K^+^-ATPase (a), SLC22A6 (b) and AQP1 immunolabeling (c) in the indicated experimental groups. The original magnification was 40×. (d–f) Whole kidney lysates were prepared from the kidneys of the sham, UUO, sham + relaxin and UUO + relaxin mice and analyzed for changes in the expression of the Na^+^/K^+^-ATPase (d), SLC22A6 (e) and AQP1 (f) mRNAs using qRT-PCR. The gene expression levels were normalized to the GAPDH levels, analyzed, and presented as means ± s.e.m; *p < 0.05 vs sham, #*p* < 0.05 vs UUO.

In light of the EMT inhibition preventing loss of TEC transporters [10,22,23], we investigated whether the relaxin can prevent loss of TEC transporters. Loss of the TEC solute and solvent transporter genes: Na^+^/K^+^-ATPase solute transporter, SLC22A6, and AQP1 (aquaporin 1) [10] were observed in the UUO. However, relaxin treatment significantly attenuated the loss of TEC transporters (Figure 6(a–c)). Consist with the results of immunohistochemistry, the results of real-time PCR indicated that UUO can inhibit the expression of TEC transporters’ mRNA. Na^+^/K^+^-ATPase, SLC22A6, and AQP1 genes were low expressed following UUO, and relaxin treatment significantly attenuated the decreasing the expression of TEC transporters genes (Figure 6(d–f)).

### Relaxin attenuated EMT-induced cell cycle arrest *in vitro*

**Figure 7.**
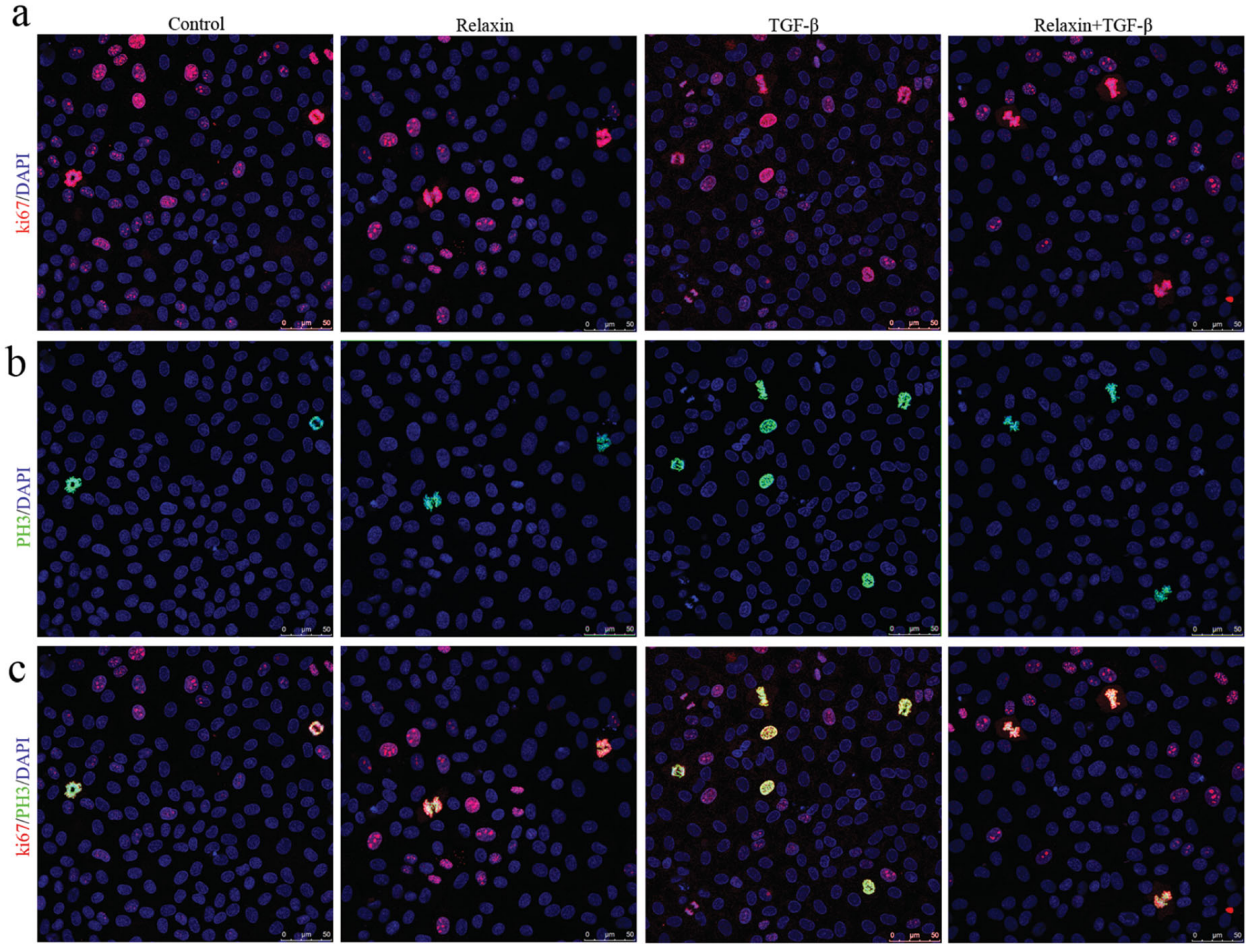
Relaxin attenuated EMT-induced cell cycle arrest *in vitro*. After 24-h incubation with a medium containing 1% FBS, the NRK52E cells were treated with blank vehicle, 10 ng/ml TGF-β1 alone, 100 ng/ml relaxin alone or both TGF-β1 and relaxin for 48 h. Representative images (three visual fields for each cell group analyzed) of Ki67 and pH-3 immunolabeling are shown. Scale bars, 50 μm.

CKD is associated with cell cycle arrest of the TECs, which arrest in the G2/M phage. TGF-β1 stimulation of TECs and EMT also results in a cell cycle arrest at the G2/M phase. We probed whether relaxin can attenuate EMT-induced cell cycle arrest *in vitro*. Consistent with previously reported, NRK-52E cells that arrest in the G2/M phase was increased after TGF-β1 stimulations. However, relaxin treatment significantly attenuated the increase of the G2/M phase NRK-52E cells after TGF-β1 stimulations (Figure 7).

## Discussion

This study explored the relaxin ameliorates renal fibrosis following UUO by attenuated EMT, prevents loss of TEC transporters, and attenuated EMT-induced cell cycle arrest. Relaxin presented an inhibition of an EMT program, leading to preservation of TECs integrity, restored proliferation, and dedifferentiation-associated repair and regeneration, and attenuated fibrosis. Therefore, we demonstrated for the first time that the novel anti-fibrosis mechanism of relaxin is inhibit the EMT through β-catenin signaling pathway thus protecting functional kidney parenchyma by facilitating cell cycle-dependent repair and regeneration of the injured kidneys.

Relaxin is a rapid-acting but safe antifibrotic that inhibits renal fibrosis in UUO [13,17]. Indeed, relaxin has anti-fibrotic effects in both *in vitro* and vivo experimental models. Garber *et al* showed that relaxin can decrease renal fibrosis and improve renal function through upregulating TGF-β levels and reducing macrophage recruitment. And other studies demonstrated that relaxin can inhibit TGF-β-induced fibroblast-myofibroblast transition and increased matrix metalloproteinases (MMPs) secretion [18,24]. However, in our study, after administration of relaxin, it was first found that the level of mesenchymal marker vimentin increased and epithelial marker E-cadherin decreased both *in vivo* and vitro. The results partly indicated that relaxin could alleviate fibrosis by inhibiting EMT. Activation of Wnt/β-catenin signaling is a common pathologic finding in a wide variety of CKD [25,26]. β-catenin is significantly increased expressed in TECs of the fibrotic kidneys, suggesting that TECs are the major targets of the canonical Wnt signaling pathway. Inhibition of Wnt/β-catenin is renoprotective, leading to alleviating renal fibrosis [25,27,28]. Numerous Wnt/β-catenin target genes (c-Myc, twist, lymphoid enhancer-binding factor 1, and snail) were induced, leading to the EMT program and renal fibrosis [29,30]. Consistent with previous studies, we found that the expression of β-catenin, snail, and the twist was increased both *in vivo* and vitro following EMT. However, relaxin treatment significantly attenuated the upregulation of β-catenin, snail, and twist, suggesting that relaxin attenuate EMT *via* Wnt/β-catenin signaling pathway.

Originally, EMT was considered to be the main mechanism by which interstitial myofibroblasts contribute to renal fibrosis [31,32]. Many studies have confirmed the occurrence of EMT in the process of renal fibrosis through a variety of models [33–35]. In addition, previous studies have confirmed that suppression of the EMT program specifically in tubular epithelial cells dramatically ameliorates renal fibrosis in multiple models, including diabetes and UUO models emphasizing the important role of EMT in renal fibrosis [36,37]. However, a recent study using an epithelial lineage-tracing coupled with reporter-tracking mesenchymal cells found than EMT contributes to the generation of only 5% of the interstitial myofibroblasts [7]. While EMT is not a simple process of morphological change. The program of EMT had a dramatic impact on the functional capabilities of TECs, reducing the expression of proteins that are fundamental for absorption and secretion activities. On the other hand, EMT could induce G2/M cell-cycle arrest, thus reducing the efficiency of normal repair of the injured kidneys [10]. Consistent with their founding, we demonstrate that the Na^+^/K^+^-ATPase solute transporter, SLC22A6, and AQP1 expression significantly decreased following UUO. Na+/K+-ATPase is an abundantly expressed protein in epithelial cells and plays an important role in kidney function. Loss Na^+^/K^+^-ATPase expression and function are accompanied by increased intracellular sodium levels and activation of MAPK signaling and might be one of the events associated with the loss of the polarized epithelial phenotype during TGF-β1 induced EMT in kidney epithelial cells and renal fibrosis [22]. and the loss of SLC22A6, the organic anion transporter, could promote the toxic accumulation of metabolites and uremic toxins [23]. Our result showed that relaxin could significantly attenuate the loss of TEC transporters, indicating that relaxin could protect the functional capabilities of TECs.

After tissue injury, the repair process involves a regeneration phase, in which injures cells are replaced by cells of the same lineage [38]. The potent capacity of TECs to proliferate and replace lost cells is crucial for repair and enables recovery from different renal injuries [39]. TECs arrested in the G2/M stage of the cell cycle after a renal injury limit the regenerative potential of the TECs and stimulate fibroblast proliferation and collagen production [11]. Conversely, reversal of the G2/M arrest attenuated the production of cytokines and fibrosis, suggesting a direct molecular link between cell cycle dysregulation in tubular epithelial cells and fibrosis. Furthermore, genome-wide unbiased transcript analysis of human kidney samples revealed that defective fatty acid oxidation in tubular epithelial cells helps to the development of renal fibrosis [40]. Since the EMT program in TECs can lead to p21-mediated G2 arrest [10], we investigated the effect of relaxin on TGF-β1-induced EMT. Consistent with previous reports, EMT induced significant G2 arrest, and relaxin treatment reversed this EMT-induced G2 arrest, suggesting that relaxin alleviates renal fibrosis by suppressing EMT and G2arrest.

However, we had inherent limitations in this study. First, we use the NRK52E to study the effect of relaxin for EMT *via* Wnt/β-catenin signaling pathways *in vitro*, rather than other TECs. Further study should investigate relaxin-regulated EMT in the TECs isolated from mice following UUO and other TECs. In addition, further verifying the role of relaxin in regulating EMT and its mechanism by *in vivo* experiments in transgenic mice and *in vitro* experiments with siRNA or inhibitors is necessary.

In conclusion, relaxin had a novel mechanism of antifibrotic through regulating the TECs *via* the Wnt/β-catenin signaling pathway. The inhibition of EMT contributes to protecting the functional capabilities of TECs and promoting the regeneration of TECs.
